# Real-World, Long-Term Outcomes of Nivolumab Therapy for Recurrent or Metastatic Squamous Cell Carcinoma of the Head and Neck and Impact of the Magnitude of Best Overall Response: A Retrospective Multicenter Study of 88 Patients

**DOI:** 10.3390/cancers12113427

**Published:** 2020-11-18

**Authors:** Takashi Matsuki, Isaku Okamoto, Chihiro Fushimi, Hideaki Takahashi, Takuro Okada, Takahito Kondo, Hiroki Sato, Tatsuya Ito, Kunihiko Tokashiki, Kiyoaki Tsukahara, Kenji Hanyu, Tatsuo Masubuchi, Yuichiro Tada, Kouki Miura, Go Omura, Michi Sawabe, Daisuke Kawakita, Taku Yamashita

**Affiliations:** 1 Department of Otorhinolaryngology, Head and Neck Surgery, Kitasato University School of Medicine, 1-15-1 Kitasato, Minami-ku, Sagamihara, Kanagawa 252-0374, Japan; matsuki@med.kitasato-u.ac.jp; 2 Department of Otorhinolaryngology, Head and Neck Surgery, Tokyo Medical University School of Medicine, 6-7-1 Nishishinjuku, Shinjuku-ku, Tokyo 160-0023, Japan; isaku@tokyo-med.ac.jp (I.O.); taku-hns@tokyo-med.ac.jp (T.O.); satohiro@tokyo-med.ac.jp (H.S.); tatsu8@tokyo-med.ac.jp (T.I.); m03075kt@tokyo-med.ac.jp (K.T.); tsuka@tokyo-med.ac.jp (K.T.); 3 Department of Head and Neck Oncology and Surgery, International University of Health and Welfare Mita Hospital, 1-4-3 Mita, Minato-ku, Tokyo 108-8329, Japan; chihiro.fushimi@iuhw.ac.jp (C.F.); kenjihanyu@iuhw.ac.jp (K.H.); masubuchi@iuhw.ac.jp (T.M.); ytada@iuhw.ac.jp (Y.T.); kmiura@iuhw.ac.jp (K.M.); 4 Department of Otorhinolaryngology, Head and Neck Surgery, Yokohama City University, 3-9 Fukuura, Kanazawa-ku, Yokohama 236-0004, Japan; htk98@yokohama-cu.ac.jp; 5 Department of Otorhinolaryngology, Head and Neck Surgery, Tokyo Medical University Hachioji Medical Center, 1163 Tatemachi, Hachioji, Tokyo 193-0998, Japan; takajin@tokyo-med.ac.jp; 6 Department of Head and Neck Oncology, National Cancer Center Hospital, 5-1-1 Tsukiji, Chuo-ku, Tokyo 104-0045, Japan; goomura@msn.com; 7 Department of Otorhinolaryngology, Head and Neck Surgery, Nagoya City University Graduate School of Medical Sciences, 1 Kawasumi, Mizuho-cho, Mizuho-ku, Nagoya, Aichi 467-8601, Japan; m.sawabe@aichi-cc.jp (M.S.); meikyo@med.nagoya-cu.ac.jp (D.K.)

**Keywords:** head and neck squamous cell carcinoma, nivolumab, long-term survival, best overall response, real-world outcome

## Abstract

**Simple Summary:**

No real-world, long-term outcomes of immunotherapy with nivolumab for recurrent or metastatic squamous cell carcinoma of the head and neck (R/M SCCHN) have yet been reported. Furthermore, the prognostic impact of the best overall response (BOR) of this therapy remains unclear. We conducted a multi-institutional cohort study of the long-term efficacy and safety of this therapy. We also evaluated the relationship between BOR and survival. Median follow-up time was 25.9 months. Median overall survival (OS) was 9.6 months, and two-year survival rate was 25.0%. Overall response rate was 18%, and disease control rate was 48%. For immune-related adverse events (irAEs), 38 irAEs were detected in 29 patients. The development of irAEs and better BOR were significantly associated with longer survival. These findings demonstrate the long-term efficacy and safety of nivolumab therapy for R/M SCCHN in a real-world setting. The magnitude of BOR and the development of irAEs might be useful surrogate markers of survival.

**Abstract:**

No real-world, long-term outcomes of immunotherapy with nivolumab for recurrent or metastatic squamous cell carcinoma of the head and neck (R/M SCCHN) have yet been reported. Furthermore, the prognostic impact of the best overall response (BOR) of this therapy remains unclear. We conducted a multi-institutional cohort study of the long-term efficacy and safety of this therapy and investigated prognostic factors associated with survival. Further, we evaluated the relationship between BOR and survival. Median follow-up time was 25.9 months. Median overall survival (OS) was 9.6 months, and two-year survival rate was 25.0%. Median progression-free survival (PFS) was 3.7 months, and two-year PFS rate was 19.6%. BOR was assessed as complete response (CR) in 6%, partial response (PR) in 13%, stable disease (SD) in 30%, and progressive disease (PD) in 52% of the patients. Overall response rate was 18%, and disease control rate was 48%. For immune-related adverse events (irAEs), 38 irAEs were detected in 29 patients. On multivariate analysis, the development of irAEs was significantly associated with better OS and PFS. Better BOR was significantly associated with longer OS and PFS. These findings demonstrate the long-term efficacy and safety of nivolumab therapy for R/M SCCHN in a real-world setting. The magnitude of BOR and the development of irAEs might be useful surrogate markers of survival.

## 1. Introduction

The prognosis of patients with recurrent or metastatic squamous cell carcinoma of the head and neck (R/M SCCHN) is poor. Treatment commonly involves platinum-based regimens, but the rate of severe adverse events with these agents is high; these include renal dysfunction and bone marrow suppression, and most patients require hospitalization.

Nivolumab, an anti-programmed death 1 (PD-1) monoclonal antibody, is an immune checkpoint inhibitor (ICI). Efficacy of nivolumab in R/M SCCHN was demonstrated in a phase 3 trial (CheckMate 141 study) [1]. Nivolumab was approved for recurrent or metastatic head and neck cancer (R/M HNC) after platinum drug administration in Japan in March 2017. Further evidence of efficacy has since been obtained [2,3,4,5,6,7,8,9,10].

We previously conducted a multicenter study of 100 patients with R/M HNC, including 88 with R/M SCCHN, treated with nivolumab from April 2017 to August 2018 [11,12,13]. The median follow-up period was 6.7 months. Efficacy was comparable to that reported previously [1,3,14]. To the best of our knowledge, although more than three years have passed since the approval of nivolumab, the only publication which reported long-term efficacy and safety of nivolumab for R/M SCCHN was a two-year long-term survival update of CheckMate 141 [2], and no real-world outcomes have been reported. Real-world data reflect a wider patient/disease background than clinical trials and are considered to conform to actual clinical practice.

Studies in lung cancer and renal cell carcinoma indicate that response to ICIs is associated with survival, [15,16] suggesting that overall response may be a useful prognostic marker of nivolumab therapy. However, the prognostic impact of response in R/M SCCHN remains unclear.

Here, we report real-world, multicenter data for the long-term efficacy and safety of nivolumab in patients with R/M SCCHN. We also assessed survival according to the best overall response (BOR) to nivolumab.

## 2. Results

### 2.1. Patient Characteristics

Eighty-eight consecutive patients with R/M SCCHN were treated with nivolumab and enrolled during the study period. Patient characteristics have been described previously [12,13] and are summarized in Table 1. Unlike the CheckMate 141 study, our study included also patients with R/M SCCHN originating in the nasopharynx, paranasal sinus, salivary gland, and external auditory canal and included patients without platinum refractoriness. The median follow-up period was 25.9 months (range, 19.0–34.4 months). At data cutoff date, 14 patients remained on nivolumab therapy. Sixty-two patients discontinued nivolumab, most commonly due to disease progression. Nine patients discontinued due to immune-related adverse events (irAEs), while two and one discontinued due to other diseases (pneumonia and atypical mycobacteriosis) and prolonged disease control (>2 years), respectively. Thirty patients received chemotherapy after nivolumab.

### 2.2. Treatment Outcomes

**Figure 1:**
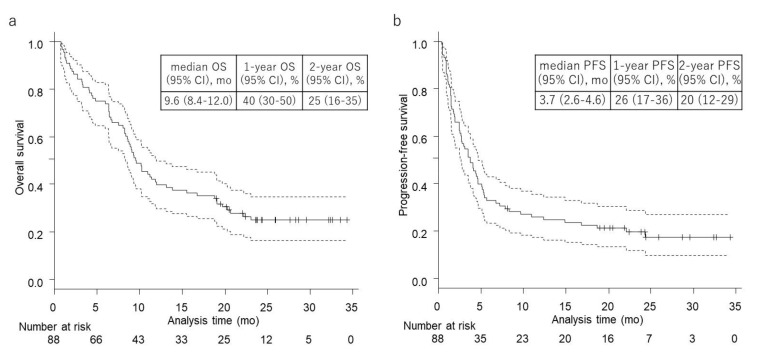
Kaplan–Meier survival curves of (**a**) overall survival (OS) and (**b**) progression-free survival (PFS) on median follow-up time of 25.9 months. (**a**) Median OS: 9.6 months (95% CI, 8.4–12.0), one-year OS: 39.8% (95% CI, 29.6–49.8), and two-year OS: 25.0% (95% CI, 16.4–34.7). (**b**) Median PFS: 3.7 months (95% CI, 2.6–4.6), one-year PFS: 26.0% (95% CI, 17.4–35.5), and two-year PFS: 19.6% (95% CI, 11.9–28.7).

Median overall survival (OS; interval between the date of first nivolumab administration and the date of death from any cause or the date of the last follow-up) was 9.6 months (95% confidence interval (95% CI), 8.4–12.0 months), and two-year survival rate was 25% (95% CI, 16–35%) (Figure 1a). Median progression-free survival (PFS; interval between the date of first nivolumab administration and the date of progressive disease (PD), the date of death from any cause, or the date of the last follow-up) was 3.7 months (95% CI, 2.6–4.6 months), and two-year PFS rate was 20% (95% CI, 12–29%) (Figure 1b).

BOR was available for all patients (Table 2). Complete response (CR) was achieved in 5 patients, partial response (PR) in 11, stable disease (SD) in 26, and PD in 46, giving an overall response rate (ORR: consisting of CR and PR) of 18% and a disease control rate (DCR: consisting of CR, PR, and SD) of 48%. Median duration from the first administration of nivolumab to the first observation of PR or CR was 80.5 days (range, 49–194 days).

### 2.3. Immune-Related Adverse Events

We summarized irAEs during the study period in Table 3. For all grades, 38 irAEs were detected in 29 patients, comprising interstitial lung disease (15%), thyroid dysfunction (9%), liver dysfunction (6%), and skin disorders (5%). For grade 3 and above, 10 irAEs occurred in 9 patients, including interstitial lung disease (6%), liver dysfunction (3%), skin disorder (1%), and enteritis (1%).

Median duration from the first administration of nivolumab to the first observation of irAEs was 75 days (range, 1–621 days). Nine patients discontinued nivolumab due to irAEs, including interstitial lung disease (six patients), liver dysfunction, enteritis, and skin disorder (one patient, respectively). We found that irAEs of grade 3 or more occurred at a median of 40 days (range, 1–266 days) from the first administration of nivolumab. Seven of 10 irAEs of grade 3 and greater were observed within 6 months, while one grade 5 interstitial lung disease occurred 266 days after the first administration of nivolumab.

### 2.4. Impact of Background Factors on Patient Survival

The impact of background factors on survival is shown in Table 4. Univariate analysis revealed that performance status (*p* < 0.001), platinum refractoriness (*p* = 0.06), and irAE (*p* = 0.065) were associated with OS. Multivariate analysis showed that performance status (*p*  <  0.001) and irAEs (*p* = 0.036) were significantly associated with OS. Likewise, performance status (*p* < 0.001), previous receipt of cetuximab (*p* = 0.042), platinum refractoriness (*p* = 0.028), and irAEs (*p* = 0.04) were associated with PFS by univariate analysis, while performance status (*p*  <  0.001) and development of irAEs (*p* = 0.023) were associated with PFS by multivariate analysis.

The median OS of patients with and without irAEs was 12.0 (95% CI 8.6–NA) and 9.1 (95% CI 6.4–10.9) months, respectively (*p* = 0.062, log-rank test). The median PFS of patients with and without irAE was 5.1 (95% CI 3.5–18.7) and 2.6 (95% CI 1.5–4.3) months, respectively (*p* = 0.037, log-rank test) (Table S1)

### 2.5. Survival According to Best Overall Response

**Figure 2:**
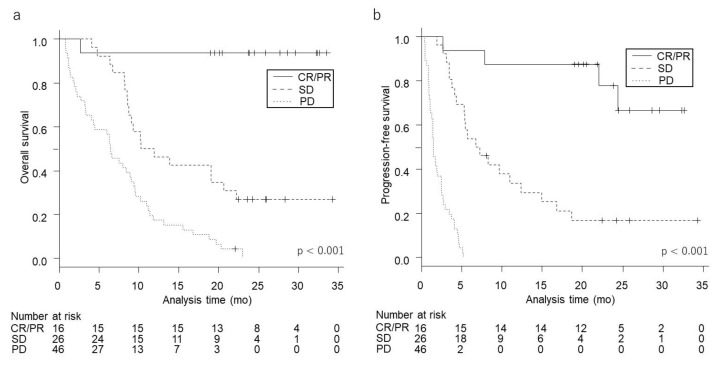
Kaplan–Meier survival curves of each best overall response for OS (**a**) and PFS (**b**). A higher magnitude of best overall response was significantly associated with longer (**a**) OS and (**b**) PFS (both, *p* < 0.001)**.**

Kaplan–Meier survival curves for OS and PFS for each magnitude of BOR are shown in Figure 2. The median and rate of one- and two-year OS and PFS for each magnitude of BOR are shown in Table 5. The median OS of patients with SD and PD was 11.1 (95% CI 8.6–20.7) and 6.4 (95% CI 3.3–9.0) months, respectively, while that of patients with CR/PR was not reached. Median PFS of SD and PD was 7.0 (95% CI 4.3–12.4) and 1.5 (95% CI 1.1–2.5) months, respectively, while that of patients with CR/PR was not reached. The rates of two-year OS of patients with CR/PR, SD, and PD were 94% (95% CI 63–99), 27% (95% CI 12–45), and 0% (95% CI, not evaluable), respectively. The rates of two-year PFS of patients with CR/PR, SD, and PD were 78% (95% CI 44–93), 17% (95% CI 5–34), and 0% (95% CI, not evaluable), respectively.

Patients whose BOR to nivolumab was PD had significantly shorter OS and PFS than those with CR/PR or SD (*p*  <  0.05, respectively).

## 3. Discussion

In this study, with a median follow-up period of 25.9 months, we demonstrated the long-term efficacy and acceptable rate of irAEs of immunotherapy with nivolumab in patients with R/M SCCHN. The magnitude of BOR was significantly associated with survival. To the best of our knowledge, this is the first study reporting real-world, long-term data on the efficacy and safety of nivolumab for R/M SCCHN and also the first to demonstrate an association between BOR to nivolumab and survival in these patients.

Limitations of this study include its retrospective design and relatively small sample size, albeit the fact that the study had a multicenter design. Accordingly, our findings might be unavoidably affected by selection bias.

Median OS (9.6 months) and median PFS (3.7 months) in our cohort were consistent with those of our previous analyses [11,12] and comparable with those in the CheckMate 141 study [1,2] (median OS of 7.7 months and median PFS of 2.0 months). Additionally, the two-year survival rate in our cohort (25%) was better than that in the CheckMate 141 study (17%). [2] ORR (18%) in our cohort was comparable to that in previous reports (13.3–26.1%), whereas DCR (48%) was better than that in the historical data (30.0–36.3%) [2,3,4,14]. This might have resulted from the fact that 28 patients in our cohort were without platinum refractoriness. Platinum refractoriness has been associated with worse prognosis in patients with R/M SCCHN [4,6]. Likewise, platinum refractoriness tended to be associated with shorter OS (hazard ratio (HR) 1.69, *p* = 0.06) and PFS (HR 1.80, *p* = 0.028) by univariate analysis in our cohort.

The incidence of irAEs (33%) in this study was similar to those in previous reports (35.9–38.1%) [4,5]. Severe irAEs are likely to occur within 6 months after the initiation of nivolumab treatment [2]; in fact, in this study, 70% of severe irAEs (Grade 3 or more) occurred within this period. In one case, however, Grade 5 interstitial lung disease occurred 8.7 months after the first administration of nivolumab, suggesting that attention to severe irAEs should continue for more than 6 months after nivolumab administration.

Interestingly, the development of irAEs has been associated with a better prognosis for nivolumab therapy [4,10,17,18,19], and our previous report showed consistent results in univariate analysis [11]. In this study, with an extended follow-up period, the development of irAE was significantly associated with better OS (HR 0.54, *p* = 0.036) and better PFS (HR 0.53, *p* = 0.023) on multivariate analysis.

Regarding the expression of programmed death-ligand 1 (PD-L1), its prognostic role for head and neck cancer has not been shown unlike for other types of carcinomas [20,21,22,23]. In R/M SCCHN patients treated with ICIs, whether overexpression of PD-L1 improves treatment outcome is still controversial [1,24]. In this study, patients with PD-L1-positive tumors tended to have longer OS and PFS, although the differences were not significant, which might be due to the fact that PD-L1 was evaluated in only 44 patients.

Nivolumab therapy is known for its unique patterns of response, such as pseudoprogression [25,26] and long-term benefit in responding patients [27,28]. The association between the magnitude of BOR and the prognosis of R/M SCCHN is therefore unclear. Consistent with other types of cancer [15,16], a higher magnitude of BOR was also associated with better survival in R/M HNSCC. Although BOR is not a predictor of efficacy before treatment, BOR might be a useful surrogate marker of survival. Whether similar results can be obtained with pembrolizumab, another anti-PD-1 antibody which is used in patients with R/M SCCHN [29], warrants investigation.

## 4. Materials and Methods

### 4.1. Patients

This study was a retrospective cohort study which enrolled patients with R/M HNC treated with nivolumab from May 2017 to August 2018 at four facilities in Japan: Kitasato University Hospital, Tokyo Medical University Hospital, Tokyo Medical University Hachioji Medical Center, and International University of Health and Welfare Mita Hospital. The medical charts of 88 consecutive patients treated with nivolumab were reviewed. All cases were histologically diagnosed as SCCHN in each facility and had been treated with platinum-based drugs before treatment with nivolumab. We excluded patients with non-SCC. Data cutoff date was 31 May, 2020. Information on patient characteristics, treatment modality, histological findings, and clinical outcomes was collected. Clinical stage was classified using the 8th edition of the TNM classification. Eastern Cooperative Oncology Group performance status (ECOG PS) was evaluated prior to administration of nivolumab. Platinum-refractory disease was defined as cancer with documented tumor progression during platinum-based treatment or recurrence within 6 months after the administration of platinum-based drugs.

The study was approved by the Institutional Ethics Review Board of each facility. Kitasato University Hospital (B20-119). Tokyo Medical University Hospital (T2020-0038). International University of Health and Welfare Mita Hospital (5-20-16). With regard to consent, patients could reject participation by opting out in response to an announcement on the institutions’ websites. This study was performed in accordance with the Declaration of Helsinki.

### 4.2. Treatment and Follow-up

Treatment and follow-up have been described in detail previously [11,12]. Nivolumab was administered at 3 mg/kg or a flat dose of 240 mg (after September 2018), the standard dose for the study period, every two weeks. After treatment initiation, CT or MRI imaging studies were performed every 4–6 weeks. Response was evaluated using Response Evaluation Criteria in Solid Tumors (RECIST) version 1.1. Clinically obvious disease progression was judged as PD regardless of imaging results, and nivolumab treatment was discontinued. After nivolumab discontinuation, additional treatment with other agents was permitted.

### 4.3. Outcomes

Endpoints were OS, PFS, and BOR. ORR and DCR were also calculated.

### 4.4. Immune-Relate Adverse Events

We assessed irAEs according to CTCAE version 5.0 up to one month after the last administration of nivolumab.

### 4.5. Statistical Analysis

OS and PFS were estimated by the Kaplan–Meier product-limit method and tested by means of two-sided log-rank tests; *p*-values for comparison among the three groups of BOR (CR/PR, SD and PD) were corrected using the Bonferroni method. Participants lost to follow-up were censored on the day of last contact. Prognostic factors, including BOR for PFS and OS, were evaluated by estimating HRs and 95% CIs using univariate and multivariate Cox proportional hazards models.

Variables adjusted in the multivariate analyses included sex (male vs. female), age (<65 vs. ≥65 years), ECOG PS (0 vs. 1–2), smoking, and alcohol consumption (ever vs. never). Platinum refractoriness (yes vs. no) and irAEs (present vs. absent) had *p*-values of less than 0.1 on univariate analysis of OS and were also included in multivariate analysis. All statistical analyses were performed using EZR [30] version 1.51 (Saitama Medical Center, Jichi Medical University, Saitama, Japan), a graphical user interface, together with the R software environment for statistical computing and graphics (The R Foundation for Statistical Computing, Vienna, Austria); *p*-values of < 0.05 were considered statistically significant.

## 5. Conclusions

In this study, we demonstrated the long-term efficacy and safety of nivolumab therapy for R/M SCCHN in a real-world setting. Furthermore, we identified a significant association between the magnitude of BOR and patient survival and also confirmed a significant positive association between the development of irAEs and patient survival. We conclude that these factors might be useful surrogate markers of survival in patients with R/M SCCHN treated with nivolumab.

## Figures and Tables

**Table 1:** Patient characteristics at baseline.

Characteristic	N = 88	(%)
Sex		
Male	71	(81)
Female	17	(19)
Age		
<65 years	39	(44)
≥65 years	49	(56)
ECOG performance status		
0	59	(67)
1	24	(27)
≥2	5	(6)
Primary site		
Nasopharynx	10	(11)
Oropharynx	19	(22)
p16-positive	8	(9)
Hypopharynx	21	(24)
Larynx	10	(11)
Oral cavity	17	(19)
Paranasal sinus	6	(7)
Salivary gland	2	(2)
External auditory canal	3	(3)
Histology		
Squamous cell carcinoma	88	(100)
Smoking		
Ever	61	(69)
Never	26	(30)
Unknown	1	(1)
Alcohol		
Ever	69	(78)
Never	17	(19)
Unknown	2	(2)
Distant metastasis		
Present	26	(30)
Absent	52	(59)
Previous exposure to cetuximab		
Yes	30	(34)
No	58	(66)
Platinum refractoriness ^a^		
Yes	28	(32)
No	60	(68)
Previous radiotherapy		
Yes	17	(19)
No	71	(81)
Previous number of chemotherapy regimens ^b^		
1	37	(42)
2	35	(40)
≥3	16	(18)
PD-L1 expression		(0)
Positive (≥1%)	37	(42)
Negative (<1%)	7	(8)
Unknown	44	(50)
Institution		(0)
TMU	41	(47)
IUHW	24	(27)
KU	15	(17)
TMU/HMC	8	(9)

^a^ Time from the final administration of platinum-based drugs until progressive disease was less than 6 months. ^b^ The category “previous number of chemotherapy regimens” includes all the chemotherapy given in the treatment of the SCCHN. Abbreviations: ECOG, Eastern Cooperative Oncology Group; TMU, Tokyo Medical University; IUWH, International University of Health and Welfare Mita Hospital; KU, Kitasato University; and TMU/HMC, Tokyo Medical University Hachioji Medical Center. Median follow-up time was 25.9 months.

**Table 2:** Best overall response.

Best Overall Response	N = 88	(%)
Complete response (CR)	5	(6)
Partial response (PR)	11	(13)
Stable disease (SD)	26	(30)
Progressive disease (PD)	46	(52)
Response rate (CR + PR)	16	(18)
Disease control rate (CR + PR + SD)	42	(48)

Abbreviations: CR, complete response; PR, partial response; SD, stable disease; PD, progressive disease.

**Table 3:** Immune-related adverse events.

Immune-Related Adverse Events	Any Grade	Grade 3–5
Interstitial lung disease	13	5
Thyroid dysfunction	8	0
Liver dysfunction	5	3
Skin disorders	4	1
Enteritis	1	1
Adrenal insufficiency	1	0
Infusion reactions	1	0
Myositis	1	0
Parathyroid dysfunction	1	0
Pituitary dysfunction	1	0
Renal dysfunction	1	0
Rheumatoid arthritis	1	0
Total	38	10
	(29 patients)	(9 patients)

**Table 4:** Univariate and multivariate analyses of background factors for overall survival and progression-free survival.

	Overall Survival				Progression-Free Survival			
Background Factors	Crude		Adjusted ^a^		Crude		Adjusted ^a^	
	HR (95% CI)	*p*-Value	HR (95% CI)	*p*-Value	HR (95% CI)	*p*-Value	HR (95% CI)	*p*-Value
Sex								
Male (Ref. Female)	1.03 (0.54–1.98)	0.922	0.54 (0.22–1.34)	0.185	1.19 (0.64–2.23)	0.581	0.64 (0.28–1.45)	0.281
Age								
≥65 years (Ref. < 65 years)	1.37 (0.83–2.24)	0.216	1.27 (0.76–2.14)	0.362	0.97 (0.61–1.56)	0.914	0.79 (0.48–1.30)	0.354
ECOG performance status								
2–3 (Ref. 0–1)	16.91 (5.69–50.32)	<0.001	20.07 (6.35–63.39)	<0.001	6.00 (2.31–15.53)	<0.001	7.87 (2.77–22.35)	<0.001
Smoking								
Ever (Ref. Never)	1.17 (0.67–2.04)	0.583	0.86 (0.42–1.75)	0.669	1.16 (0.69–1.94)	0.587	0.86 (0.45–1.66)	0.660
Alcohol consumption								
Ever (Ref. Never)	1.51 (0.77–2.98)	0.230	1.64 (0.67–4.03)	0.278	1.61 (0.84–3.08)	0.149	1.60 (0.71–3.60)	0.257
Distant metastasis								
Present (Ref. Absent)	0.94 (0.57–1.55)	0.818			0.95 (0.59–1.53)	0.844		
Previous exposure to cetuximab								
Yes (Ref. No)	1.43 (0.84–2.45)	0.188			1.71 (1.02–2.88)	0.042		
Platinum refractoriness								
Yes (Ref. No)	1.69 (0.98–2.91)	0.060	1.55 (0.80–3.01)	0.196	1.80 (1.07–3.05)	0.028	1.78 (0.96–3.30)	0.067
Previous radiotherapy								
Yes (Ref. No)	1.05 (0.56–1.96)	0.883			0.83 (0.46–1.50)	0.539		
Previous number of chemotherapy regimens								
two or more (Ref. 1)	1.05 (0.63–1.73)	0.860			1.12 (0.70–1.80)	0.640		
PD-L1 expression								
Positive (Ref. Negative)	0.58 (0.25–1.35)	0.203			0.50 (0.21–1.19)	0.117		
p16 expression								
Positive (Ref. Negative or Unknown)	0.66 (0.28–1.53)	0.332			0.76 (0.35–1.66)	0.490		
Immune-related adverse events								
Yes (Ref. No)	0.60 (0.35–1.03)	0.065	0.54 (0.31–0.96)	0.036	0.59 (0.35–0.98)	0.040	0.53 (0.31–0.92)	0.023

^a^ Adjusted by sex, age, performance status, smoking, alcohol consumption, platinum refractoriness, and immune-related adverse events. Abbreviations: HR, hazard ratio; CI, confidence interval.

**Table 5:** Relationship between best overall response and survival.

			**Overall Survival**								
									Crude		
	N = 88	(%)	median (months)	(95% CI)	1-year OS (%)	(95% CI)	2-year OS (%)	(95% CI)	HR	(95% CI)	*p*-value
CR/PR	16	(18)	NR		94	(63–99)	94	(63–99)	0.02	(0.003–0.15)	<0.001
SD	26	(30)	11.1	(8.6–20.7)	46	(27–64)	27	(12–45)	0.37	(0.21–0.65)	0.001
PD	46	(52)	6.4	(3.3–9.0)	17	(8–30)	0	(NA-NA)	1 (reference)		
			**Progression-Free Survival**								
									Crude		
	N = 88	(%)	median (months)	(95% CI)	1-year PFS (%)	(95% CI)	2-year PFS (%)	(95% CI)	HR	(95% CI)	*p*-value
CR/PR	16	(18)	NR	(22.1-NA)	88	(59–97)	78	(44–93)	0.02	(0.005–0.06)	<0.001
SD	26	(30)	7.0	(4.3–12.4)	34	(16–52)	17	(5–34)	0.10	(0.05–0.22)	<0.001
PD	46	(52)	1.5	(1.1–2.5)	NA	(NA-NA)	0	(NA-NA)	1 (reference)		

Abbreviations: NA, not available; NR, not reached.

## Supplementary Materials

The following are available online at https://www.mdpi.com/2072-6694/12/11/3427/s1, Table S1: Relationship between immune-related adverse events and survival.

## References

[1] Ferris R.L., Blumenschein G., Fayette J., Guigay J., Colevas A.D., Licitra L., Harrington K., Kasper S., Vokes E.E., Even C. (2016). Nivolumab for Recurrent Squamous-Cell Carcinoma of the Head and Neck. N. Engl. J. Med..

[2] Ferris R.L., Blumenschein G., Fayette J., Guigay J., Colevas A.D., Licitra L., Harrington K.J., Kasper S., Vokes E.E., Even C. (2018). Nivolumab vs investigator’s choice in recurrent or metastatic squamous cell carcinoma of the head and neck: 2-year long-term survival update of CheckMate 141 with analyses by tumor PD-L1 expression. Oral Oncol..

[3] Kiyota N., Hasegawa Y., Takahashi S., Yokota T., Yen C.J., Iwae S., Shimizu Y., Hong R.L., Goto M., Kang J.H. (2017). A randomized, open-label, Phase III clinical trial of nivolumab vs. therapy of investigator’s choice in recurrent squamous cell carcinoma of the head and neck: A subanalysis of Asian patients versus the global population in checkmate 141. Oral Oncol..

[4] Hori R., Shinohara S., Kojima T., Kagoshima H., Kitamura M., Tateya I., Tamaki H., Kumabe Y., Asato R., Harada H. (2019). Real-World Outcomes and Prognostic Factors in Patients Receiving Nivolumab Therapy for Recurrent or Metastatic Head and Neck Carcinoma. Cancers.

[5] Ueki Y., Takahashi T., Ota H., Shodo R., Yamazaki K., Horii A. (2020). Predicting the treatment outcome of nivolumab in recurrent or metastatic head and neck squamous cell carcinoma: Prognostic value of combined performance status and modified Glasgow prognostic score. Eur. Arch. Otorhinolaryngol..

[6] Inoue H., Yokota T., Hamauchi S., Onozawa Y., Kawakami T., Shirasu H., Notsu A., Yasui H., Onitsuka T. (2020). Pre-treatment tumor size impacts on response to nivolumab in head and neck squamous cell carcinoma. Auris Nasus Larynx.

[7] Suzuki C., Kiyota N., Imamura Y., Rikitake J., Sai S., Koyama T., Hyogo Y., Nagatani Y., Funakoshi Y., Toyoda M. (2020). Effect of tumor burden and growth rate on treatment outcomes of nivolumab in head and neck cancer. Int. J. Clin. Oncol..

[8] Niwa K., Kawakita D., Nagao T., Takahashi H., Saotome T., Okazaki M., Yamazaki K., Okamoto I., Hirai H., Saigusa N. (2020). Multicentre, retrospective study of the efficacy and safety of nivolumab for recurrent and metastatic salivary gland carcinoma. Sci. Rep..

[9] Fushimi C., Okamoto I., Matsuki T., Masubuchi T., Okada T., Sato H., Tsukahara K., Kondo T., Yamashita T., Hanyu K. (2020). Salvage Chemotherapy After Nivolumab for Recurrent or Metastatic Head and Neck Carcinoma. Anticancer Res..

[10] Matsuo M., Yasumatsu R., Masuda M., Toh S., Wakasaki T., Hashimoto K., Taura M., Uchi R., Nakagawa T. (2020). Relationship between immune-related adverse events and the long-term outcomes in recurrent/metastatic head and neck squamous cell carcinoma treated with nivolumab. Oral Oncol..

[11] Okamoto I., Sato H., Kondo T., Koyama N., Fushimi C., Okada T., Miura K., Matsuki T., Yamashita T., Omura G. (2019). Efficacy and safety of nivolumab in 100 patients with recurrent or metastatic head and neck cancer—A retrospective multicentre study. Acta Otolaryngol.

[12] Matsuki T., Okamoto I., Fushimi C., Sawabe M., Kawakita D., Sato H., Tsukahara K., Kondo T., Okada T., Tada Y. (2020). Hematological predictive markers for recurrent or metastatic squamous cell carcinomas of the head and neck treated with nivolumab: A multicenter study of 88 patients. Cancer Med..

[13] Kondo T., Okamoto I., Sato H., Koyama N., Fushimi C., Okada T., Masubuchi T., Miura K., Matsuki T., Yamashita T. (2020). Age-based efficacy and safety of nivolumab for recurrent or metastatic head and neck squamous cell carcinoma: A multicenter retrospective study. Asia Pac. J. Clin. Oncol..

[14] Nishikawa D., Suzuki H., Koide Y., Beppu S., Kadowaki S., Sone M., Hanai N. (2018). Prognostic Markers in Head and Neck Cancer Patients Treated with Nivolumab. Cancers.

[15] Antonia S.J., Borghaei H., Ramalingam S.S., Horn L., De Castro Carpeño J., Pluzanski A., Burgio M.A., Garassino M., Chow L.Q.M., Gettinger S. (2019). Four-year survival with nivolumab in patients with previously treated advanced non-small-cell lung cancer: A pooled analysis. Lancet Oncol..

[16] Ishihara H., Takagi T., Kondo T., Tachibana H., Fukuda H., Yoshida K., Iizuka J., Kobayashi H., Okumi M., Ishida H. (2019). Correlation between the magnitude of best tumor response and patient survival in nivolumab therapy for metastatic renal cell carcinoma. Med. Oncol..

[17] Rogado J., Sánchez-Torres J.M., Romero-Laorden N., Ballesteros A.I., Pacheco-Barcia V., Ramos-Leví A., Arranz R., Lorenzo A., Gullón P., Donnay O. (2019). Immune-related adverse events predict the therapeutic efficacy of anti-PD-1 antibodies in cancer patients. Eur. J. Cancer.

[18] Teraoka S., Fujimoto D., Morimoto T., Kawachi H., Ito M., Sato Y., Nagata K., Nakagawa A., Otsuka K., Uehara K. (2017). Early Immune-Related Adverse Events and Association with Outcome in Advanced Non-Small Cell Lung Cancer Patients Treated with Nivolumab: A Prospective Cohort Study. J. Thorac. Oncol..

[19] Haratani K., Hayashi H., Chiba Y., Kudo K., Yonesaka K., Kato R., Kaneda H., Hasegawa Y., Tanaka K., Takeda M. (2018). Association of Immune-Related Adverse Events With Nivolumab Efficacy in Non-Small-Cell Lung Cancer. JAMA Oncol..

[20] Ma G., Deng Y., Jiang H., Li W., Wu Q., Zhou Q. (2018). The prognostic role of programmed cell death-ligand 1 expression in non-small cell lung cancer patients: An updated meta-analysis. Clin. Chim. Acta.

[21] Wang Z., Peng S., Xie H., Guo L., Cai Q., Shang Z., Jiang N., Niu Y. (2018). Prognostic and clinicopathological significance of PD-L1 in patients with renal cell carcinoma: A meta-analysis based on 1863 individuals. Clin. Exp. Med..

[22] Zhang M., Sun H., Zhao S., Wang Y., Pu H., Wang Y., Zhang Q. (2017). Expression of PD-L1 and prognosis in breast cancer: A meta-analysis. Oncotarget.

[23] Troiano G., Caponio V.C.A., Zhurakivska K., Arena C., Pannone G., Mascitti M., Santarelli A., Lo Muzio L. (2019). High PD-L1 expression in the tumour cells did not correlate with poor prognosis of patients suffering for oral squamous cells carcinoma: A meta-analysis of the literature. Cell Prolif..

[24] Jia Y.Q., Yang B., Wen L.L., Mu W.X., Wang Z., Cheng B. (2019). Prognostic value of immune checkpoint molecules in head and neck cancer: A meta-analysis. Aging.

[25] Queirolo P., Spagnolo F. (2017). Atypical responses in patients with advanced melanoma, lung cancer, renal-cell carcinoma and other solid tumors treated with anti-PD-1 drugs: A systematic review. Cancer Treat. Rev..

[26] Lauber K., Dunn L. (2019). Immunotherapy Mythbusters in Head and Neck Cancer: The Abscopal Effect and Pseudoprogression. Am. Soc. Clin. Oncol. Educ. Book.

[27] Ferrara R., Pilotto S., Caccese M., Grizzi G., Sperduti I., Giannarelli D., Milella M., Besse B., Tortora G., Bria E. (2018). Do immune checkpoint inhibitors need new studies methodology?. J. Thorac. Dis..

[28] Vivot A., Créquit P., Porcher R. (2019). Use of Late-Life Expectancy for Assessing the Long-Term Benefit of Immune Checkpoint Inhibitors. J. Natl. Cancer Inst..

[29] Burtness B., Harrington K.J., Greil R., Soulières D., Tahara M., de Castro G., Psyrri A., Basté N., Neupane P., Bratland Å. (2019). Pembrolizumab alone or with chemotherapy versus cetuximab with chemotherapy for recurrent or metastatic squamous cell carcinoma of the head and neck (KEYNOTE-048): A randomised, open-label, phase 3 study. Lancet.

[30] Kanda Y. (2013). Investigation of the freely available easy-to-use software ‘EZR’ for medical statistics. Bone Marrow Transplant..

