# Supplementary material for: Real-World, Long-Term Outcomes of Nivolumab Therapy for Recurrent or Metastatic Squamous Cell Carcinoma of the Head and Neck and Impact of the Magnitude of Best Overall Response: A Retrospective Multicenter Study of 88 Patients
Source: Cancers (Basel). 2020 Nov 18;12(11):3427. doi: 10.3390/cancers12113427 (PMC7699139; doi:10.3390/cancers12113427)
Supplement: Supplementary file 1 [file cancers-12-03427-s001.pdf]

Supplementary Materials

Real-World, Long-Term Outcomes of Nivolumab Therapy for Recurrent or Metastatic Squamous Cell Carcinoma of the Head and Neck and Impact of the Magnitude of Best Overall Response: A Retrospective Multicenter Study of 88 Patients

Takashi Matsuki, Isaku Okamoto, Chihiro Fushimi, Hideaki Takahashi, Takuro Okada, Takahito Kondo, Hiroki Sato, Tatsuya Ito, Kunihiko Tokashiki, Kiyoaki Tsukahara, Kenji Hanyu, Tatsuo Masubuchi, Yuichiro Tada, Kouki Miura, Go Omura, Michi Sawabe, Daisuke Kawakita and Taku Yamashita

Table S1. Relationship between immune-related adverse events and survival

**immune-related adverse events**			Overall survival						
	N = 88	(%)	median (months)	(95% CI)	1-year OS (%)	(95% CI)	2-year OS (%)	(95% CI)	p-value [a]
irAE (+)	29	(33)	12.0	(8.6 -NA)	48	(30 -65)	37	(20 -54)	0.062
irAE (-)	59	(67)	9.1	(6.4 -10.9)	36	(24 -48)	19	(10 -30)	

**immune-related adverse events**			Progression-free survival						
	N = 88	(%)	median (months)	(95%CI)	1-year PFS (%)	(95%CI)	2-year PFS (%)	(95%CI)	p-value
irAE (+)	29	(33)	5.1	(3.5 -18.7)	38	(21 -55)	26	(11 -43)	0.037
irAE (-)	59	(67)	2.6	(1.5 -4.3)	20	(11 -31)	16	(8 -27)	

[a] log-rank test

Abbreviations: irAE, immune-related adverse event; NA, not available; CI, confidence interval
